# Abstract of the Proceedings of the Buffalo Medical Association, Reported by the Secretary

**Published:** 1859-03

**Authors:** Austin Flint


ART. V.—*Abstract of Proceedings of the Buffalo Medical Association.*

TUESDAY EVENING, Feb. 1, 1859.

The Association met.

Present—The President, Dr. Wyckoff in the chair; Drs. Newman, Jansen, Butler, Hawley, Whitney, Treat, Wilcox, Congar, Miner, and Flint, Jr.

The minutes of the last meeting were read and approved.

Dr. Mead was elected a member of the Association, on his complying with the usual formalities.

Dr. MINER read the following paper upon Tetanus:

Since the Association made it my duty to report upon the frequency of tetanus, and its proper treatment, I have collected some facts upon the subject; though, by no means, do I pretend to speak upon the general subject of tetanus, or discuss its many points of interest and importance.

I find that actual statistics upon the point of frequency in our county, are not within the reach of any research. Some of our older physicians have met, in their own and others' practice, three or four cases, while others, of equal experience, have never met a single case. In the absence of more definite knowledge, and to give some idea of the frequency of the disease in our climate, I propose to draw from the United States mortality statistics of the census of 1850. I will not detain you with a detail of all the States; a few will serve to show its comparative frequency in the colder and warmer climates.

| States. | No. Deaths. |
|---|---|
| In Maine, | 1 |
| " New Hampshire, | 2 |
| " Vermont, | 1 |
| " Massachusetts, | 6 |
| " Rhode Island, | 3 |
| " Connecticut, | 6 |
| " New York, | 23 |
| " New York city, State N. Y., | 5 |

While taking the southern portion of the country:

| States. | No. Deaths. |
|---|---|
| In Louisiana, | 215 |
| " Kentucky, | 38 |
| " Mississippi, | 60 |
| " Georgia, | 23 |
| " Alabama, | 6 |

In the north-western states and territories, no cases are recorded as dying from this cause. It is not to be supposed that this record is very correct; since the regulations concerning the registry of deaths is very imperfect, and in many places wholly unknown.

The number of deaths is so near the number of cases, that could we know the actual number of deaths, we should be able to judge with sufficient accuracy of the frequency of the disease.

Of the whole number in the United States, 694, there were 367 under one year of age, this including the negro children, who die of trismus nascentium, the disease affecting infants soon after birth; said to be very common in the West Indies, and some of the southern states.

In speaking of the frequency of the disease, I have already intimated that a large majority of the cases terminate fatally. This disease, so frequently affecting negro children, is said, rarely, if ever, to terminate favorably. The disease in our climate, produced by some injury, constituting the traumatic tetanus, is fatal, except in rare instances, where the disease is not fully developed, where the paroxysms of spasm are mild and infrequent, and where it partakes of the chronic form, as it has been quite inappropriately termed, appearing late after the injury. In this, I know I am exposed to attack, by physicians ambitious to report some wonderful remedy or new plan of treatment which they have invented or applied, and which has thus far given evidence of great promise. It is not so dangerous to be attacked in our opinions by doctors, who lose no cases of tetanus, or croup, or similar diseases, usually fatal in other hands, as to be doctored by them. That the disease does sometimes, in the milder cases, terminate favorably, there can be no doubt; that it will do this, independently of any medication, may be also abundantly demonstrated.

I do not desire to say that medicines exert no mitigating influence, and that all cases have a direction, which proper medication cannot, and does not, change, but rather that the acute cases are, in great majority, fatal, while those of milder character do sometimes recover.

Perhaps I shall be pardoned for digressing sufficiently from the rule under which I write, to speak briefly of the pathology of tetanus, since treatment cannot be properly considered independently. This will open up a field for inquiry and research, so extensive, I fear I shall be led from my purpose, and trespass upon your time and patience.

The most common view of that form of tetanus which follows some wounds, is, no doubt, that the exaltation of spinal nerve force, upon which the tenanic phenomena depend, is the result of continuous irritation of some afferent nerve at a remote point. Of this view, Dr. Marshall Hall, is, perhaps, the most distinguished exponent. Many facts are cited, which strongly favor it. On the other hand, there are many difficulties it does not meet. We have no proof that mere local irritation of a nerve is capable of producing such a condition of the spinal cord, as that which we see in this remarkable disorder.

Some pathologists have, therefore, been led to suppose that this condition may possibly be brought about, not through the nerve at all, but through the blood, and may be due, in fact, to the development and introduction into that fluid of some morbid poison. These are the two theories which divide medical opinion on this subject. Wm. Bull, upon the supposition of blood poison, and with the view that the poison resembled strichnine in its physiological action, in the summer of 1853, endeavored to put this question to the test of experiment. The method followed was identical with that proposed by Dr. Marshall Hall for the detection of strichnine.

An aqueous solution of the spinal cord of a man, who had died of tetanus, was carefully made. A portion of blood was also prepared from the same man; into each a frog was placed, as in a bath. Alcoholic extracts from the same cord were also injected into the cellular tissue of other frogs, and introduced under the integument; although the results were entirely negative, yet this question of blood poison is still open for consideration.

Whatever might have been the fate of the doctor's frogs, it would not seem capable of giving us any proof upon the question of blood-poisoning in tetanus, had they died suddenly, and in spasms; and this question, no less difficult to decide, presents itself: Was it tetanic poison, or dead animal poison? I can readily conceive that these wounds, though often apparently healthy, are yet the laboratory, where chemical changes are taking place, and poisonous compounds eliminated, resembling those morbid products, which are known to exist soon after the extinction of life; that there is, indeed, a partial death of the parts, and that it is subject, in more or less degree, to chemical changes, which are supposed to produce, in animal decompositions, the most virulent and fatal poisons of which we have any knowledge.

Objections, I am aware, may very properly be urged: that wounds heal, and are, to all appearance, healthy, before any symptoms of tetanus appear; that we have no known products of animal decomposition, which produce anything of tetanic convulsions; that the nervous system seems alone to suffer; and other objectious of equal weight and importance; but none, so far as I am aware, unanswerable, or fatal to this hypothesis, which certainly is not capable of demonstration, and even may be regarded by many as not the most probable.

"Considering how signally our present modes of practice fail in this dreadful malady, it is not creditable, if no efforts be made to gain some deeper insight into the nature of this disease, to go on with a repetition of the old remedies, while one after another of our patients die under our care, and not at the same time do all in our power to gain some new and clearer views of the essence of the disease, is not the part of men, whose office is to save life, and who are earnest in their vocation. New theories should be advanced; more searching methods instituted, and deeper research made, since common observation has taught us all it can teach."— *Wm. Bull.*

*Post-mortem* appearances fail in throwing any very strong light upon us in this darkness. The nerves leading to, or from, an injury which has been followed by tetanus, are often in no way effected other than in wounds, followed by no such results. The spinal cord is sometimes affected with irritation, congestion, or effusion. That this is not the *cause* of the disease, but only the *results* or *effects*, seems highly probable.

The wounds themselves, however, are often unhealthy, and the source of unhealthy discharges. The only cases which have fallen under my observation were excellent examples and illustrations of this fact. In one, there was a slough of the integument, and final separation of the bones of the finger; in the other, Dr. Wyckoff and myself found a discolored and changed appearance in the cellular tissue, immediately surrounding the wound, without appearance of healthy inflammation and ulceration, though the splinter of wood, which was the cause, remained eight or nine days, when it was removed by Dr. Wyckoff, at his first visit.

Before speaking of the remedies useful in this disease, I will say that, perhaps, there is no disease in which curative and preventative measures have been more extensively used than tetanus, and none in which greater difficulty existed in determining what influence they exerted. With our ignorance of the pathology of tetanus in consideration, we are not surprised that the treatment in many cases is strictly empirical. Neither do we wonder that the most opposite remedies have been occasionally used with apparent success; and, each, in turn, rejected, as unworthy of confidence. The success of our remedies, when applied in the treatment of diseases, which naturally terminate fatally, is mcre easily estimated. In the influence of remedies in malignant diseases, few are mistaken.

In our ignorance of the inner nature of this disease, and any remedies, which apply as specifics, we must consider the symptoms, constituting, as they do, plain indications for treatment.

It is of the greatest importance, first to remove any exciting cause; but amputations, and all severe operative measures should be avoided; it has never been justified by favorable results, though formerly extensively adopted.

To abate or control the tetanic spasm, all the narcotics and antispasmodics have been perseveringly and faithfully tried. More recently, chloroform and ether have been claimed as most worthy of confidence.

While I deny its ability to prevent the regular return of the spasm in all cases, yet I am ready to admit, that at present, it does really promise us very much. How much, is yet to be determined. I have found, that though a patient is fully under the influence of chloroform, the spasms recur at about the same intervals; the depressing influences of both the disease and chloroform are conjoined, and it still remains to be satisfactorily shown, that anæsthetics are serviceable and safe.

It must be certain that much depends, in these cases, upon the faithful administration of *food*, *stimulants*, *tonics*, and *anodynes*. With their timely aid, cases which show any opportunity for their effects, will be greatly sustained, and chances for favorable termination increased.

Dr. Butler reported a case of *Purpura Hæmorragica* occurring in a nursing child six months old. The kidneys were shown the Association. The history was as follows:

Dr. Butler saw the child early in January; it was pale and waxen in appearance, having a branny eruption on the lower part of the neck, extending to between the shoulders. A simple lotion was ordered, with cod liver oil internally.

On January 15th his attention was called to the eruption on the neck, which had assumed a scarlet appearance, and was inclined to bleed.

Applied mr. tinct. iron, with iron internally.

January 20. Attention called to the child on account of diarrhœa. Hydrag cum creta improved the bowels. The mother called attention to large bruises on both sides of the head, and posteriorly, on the occipital bone, saying the child had fallen from the bed during the night, and supposed it was due to that. An examination of the body revealed spots on the arms, back, legs and foot, of bluish color, resembling bruises.

Dr. Rochester saw the child, and agreed with Dr. Butler in pronouncing it, unmistakably a case of purpura hæmorragica.

*Treatment.* Gallic acid gr. j, with brandy every four hours. This seemed to make a feeble impression for a few days, but the child gradually sank, and died January 28.

*Post-mortem*, four days after. No decomposition or smell apparent; external appearance, as during life, pale, with blue and purplish spots on the head, chin, neck, arms, fingers, legs, feet, and on the back, near spine; none on abdomen, or thighs; the abdomen and chest only examined; the skin cut dry, not a drop of blood exuded; lungs healthy, though considerably collapsed; heart and liver healthy; gall bladder, full of greenish yellow fluid; spleen, perhaps, slightly enlarged, of a dark purple color, no spots, normal in appearance. Found both kidneys diseased, being much engorged with blood in the centre, or pelvis, the left more than the right. The vessels at the apex, and around the left, much congested.

*Remarks.* This case is, perhaps, a little peculiar, beginning in a scurfy desquamation on the neck. Purpura hæmorrhagica is described as generally commencing on the legs. After the discharge of blood began it did not cease; no cough, no blood oozed from mouth, gums, or vagina. Eberle, in his practice, mentions having seen but one case, occurring in a child seven years of age, recovered. "Dr. Habershon saw five cases in his practice, three of them fatal. Found the spleen diseased in all the cases, of a dull red color, studded with pale yellow spots." Condie remarks, that it is of more frequent occurrence before than after puberty; and attacks those in badly ventilated rooms, badly fed, &c.

Dr. Jansen reported a case of gonorrhœa in a male child, four years of age. He had a discharge, which was apparently gonorrheal, with swelling of the parts, and phymosis. Cold dressings were first applied without benefit. Warm water dressings were then substituted, which relieved the inflammation. The patient recovered in a few days. Dr. Jansen enquired if any members of the Association had seen cases of gonorrhœa at this early age.

Dr. Treat had seen a discharge in female children which closely resembled gonorrhœa.

Dr. Whitney had seen an undoubted case of gonorrhœa in a child four years old.

Dr. Flint, Jr., remarked that there was a disease of female children, which was not very uncommon, which consisted in a discharge from the genital organs, much resembling gonorrhœa. Authors stated that it was difficult, in some cases, to distinguish between this infantile leucorrhœa and gonorrhœa. He had been called upon to treat a case, which was supposed to be a gonorrhœa, contracted from a boy, who was accused of having violated the patient, a little negro girl, six years old. The boy was indicted for rape, and in his testimony, Dr. Flint stated that doubt would arise in the case, and that it would be almost impossible to pronounce, with certainty, that the disease was gonorrhœal. The boy was examined about ten days after the alleged violation, and was found healthy. The other circumstances of the case, however, were sufficient to convict the prisoner. This case yielded in the course of three or four weeks.

Dr. Newman mentioned that there had been a discussion of this subject in the English journals some time ago. Parties had been arrested on suspicion of having committed rape upon children, and were nearly convicted, upon the testimony of the children themselves, when medical investigation brought to light the fact, that infantile leucorrhœa was an epidemic in that neighborhood. He thought that the readiness with which the disease yielded to treatment, was a valuable diagnostic sign; cases of leucorrhœa yielding in a few days to simple measures, and gonorrhœa continuing for weeks.

Dr. Wilcox had seen three cases of infantile gonorrhœa. In one case, the eye was lost from gonorrhœal opthalmia, and the patient had gonorrhœal rheumatism. In one of the remaining cases, the patient also had gonorrhœal rheumatism; and in the other, he had every reason to be confident in his diagnosis.

Dr. Butler mentioned an ordinary case of gonorrhœa, in which the discharge persisted for a long time, but finally stopped, when the irritation of the flannel shirt was removed; the discharge was, he thought, kept up by the contact of the red flannel.

Dr. Wilcox presented to the Association a small iron chain, ten inches in length, which had been accidentally swallowed by an infant, and was passed *per anum* on the following day without inconvenience.

Dr. Miner mentioned the case of a person who had swallowed an ordinary pin, which came out at the knee six months afterwards.

Drs. Rathbun and Whittaker were proposed for membership.

The Association then adjourned.

AUSTIN FLINT, Jr., M. D.,
Secretary.

